# Effects of Cerebellar Repetitive Transcranial Magnetic Stimulation at Different Frequencies on Working Memory: An EEG Study

**DOI:** 10.1111/cns.70491

**Published:** 2025-07-16

**Authors:** Bo Song, Minjie Tian, Tong Wang, Xixi Wang, Xing Ye, Qun Yao, Jingping Shi, Kuiying Yin

**Affiliations:** ^1^ Department of Neurology The Affiliated Brain Hospital of Nanjing Medical University Nanjing China; ^2^ Nanjing Research Institute of Electronic Technology Nanjing China

**Keywords:** brain network, cerebellum, event‐related potentials, neural oscillatory activity, repetitive transcranial magnetic stimulation

## Abstract

**Background:**

The cerebellum serves as an important target for non‐invasive cognitive regulation because of its involvement in diverse cognitive processes via cerebro‐cerebellar circuits. However, the efficiency of repetitive transcranial magnetic stimulation (rTMS) in different cognitive behavior protocols remains elusive.

**Objective:**

To investigate changes in working memory and behavioral performance after different cerebellar rTMS to provide objective neurobiological guidance for optimizing cerebellar TMS parameters.

**Methods:**

A total of 75 healthy adult volunteers underwent rTMS at various frequencies (1, 5, 10, and 20 Hz) or received sham stimulation. Electroencephalogram recordings were captured in the resting state and 2‐back working memory tasks, both before and after stimulation. Behavioral results, event‐related potential (ERP) waveforms and spectrum, and brain network topology changes were compared among the groups and rTMS conditions.

**Results:**

Behavioral results significantly differed between the 5 Hz stimulation and sham groups. Analysis of ERP data suggested that 5 Hz stimulation significantly increased P150 amplitude compared with sham. Time‐frequency analysis showed enhanced θ and α oscillations after 5 Hz stimulation, with α oscillations significantly higher than those in sham stimulation. Resting state brain network analysis demonstrated increased θ band global efficiency and whole brain local efficiency after 5 Hz stimulation. In contrast, 20 Hz stimulation only reduced the shortest path length. In the 5 Hz stimulation group, P150 amplitude and α oscillation were significantly correlated with the reaction time.

**Conclusion:**

Taken together, rTMS at 5 Hz applied to the cerebellar Crus II region significantly enhances neural oscillatory activity and improves working memory.

## Graphical abstract

Our study is the first to apply rTMS across five frequency bands to the cerebellum, revealing that low‐frequency TMS does not significantly affect working memory. In contrast, 5 Hz rTMS significantly enhances working memory performance and improves the parameters of resting‐state brain networks, emphasizing the role of cerebellar rTMS in cognitive regulation.

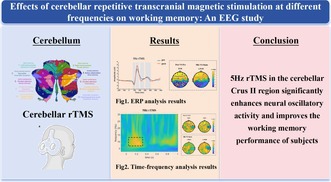

## 1 Introduction

The cerebellum has long been recognized for its role in regulating motor functions; however, its contributions to non‐motor functions have been traditionally underestimated. The cerebellum plays regulatory roles in higher cognitive activities, such as memory, learning, executive function, and emotion processing [1]. The non‐motor functions appear to originate from the specialized neuroanatomy of cerebellar lobules VI to VIII, particularly through the dense reciprocal connections of crus I/II with both cortical association areas and subcortical nuclei [2]. Notably, the closed‐loop circuitry linking the dorsolateral prefrontal cortex with cerebellar crus subregions supports executive control mechanisms and working memory operations [3]. Neuroimaging investigations have further demonstrated that cerebellar injuries induce multi‐domain cognitive impairments by disrupting global network connectivity, particularly reducing the efficiency of information integration across distributed neural systems [4]. These converging findings position the cerebellum as a promising neuromodulation target for enhancing cognitive functions, particularly working memory capacity.

Transcranial magnetic stimulation (TMS) is an effective, non‐invasive tool that modulates cortical activity [5, 6, 7, 8]. It has been widely used to investigate and treat neuropsychiatric diseases, such as depression, Alzheimer's disease (AD), and stroke [9, 10, 11]. TMS applied to specific cerebral regions improves cognitive function and may serve as an auxiliary therapy strategy [12, 13]. According to the International Federation of Clinical Neurophysiology and the Consensus of European Experts, multi‐target repetitive TMS (rTMS) strategies may provide greater benefits for cognitive rehabilitation because of the limited regulatory efficiency of a single target area in the cerebral cortex [6]. Therefore, more efficient therapeutic targets are needed for cognitive regulation. Previously, after 4 weeks of applying rTMS at 5 Hz to the bilateral cerebellar crus II, patients with AD exhibited significantly improved global cognitive function, memory, attention, visuospatial skills, and executive function, with these effects persisting up to 3 months after the intervention [14]. These results support cerebellar TMS as a promising therapeutic strategy for cognitive rehabilitation. However, because of structural differences between the cerebellum and cerebrum, stimulation parameters effective for cerebral regions may be unsuitable for the cerebellum. In light of limited studies on cerebellar stimulation, the regulatory efficiency of cerebellar TMS at different frequencies on cognition warrants investigation.

Working memory is a high‐level cognitive ability originating from interactions among attention, short‐term retention, and information manipulation [15]. It is conducted by the coordinated activation of numerous brain regions, including the cerebellum [16]. The N‐back task is widely used to investigate working memory [17], with performance under varying working memory loads determined by the reaction time and accuracy. TMS interventions significantly improve n‐back working memory performance and enhance electrophysiological activity [18, 19].

This study aims to investigate the effects of different rTMS frequencies applied to the right cerebellar Crus II region on the neurophysiology and behavioral performance of working memory. Additionally, it aims to explore the potential neural mechanisms through which TMS improves cognitive function to provide optimized parameters for cerebellar modulation and enhance cognitive function.

## 2 Materials and Methods

### 2.1 Study Participants

This single‐centered, randomized, controlled study was conducted between January 2022 and December 2023. A total of 75 healthy volunteers were recruited (average age 24.2 ± 1.2 years; 35 women). The participants had an average of 16.5 ± 2.3 years of education. The inclusion criteria were as follows: (1) Mini‐Mental State Examination (MMSE), Montreal Cognitive Assessment (MoCA), and Mini International Neuropsychiatric Interview (MINI) scores within the normal range [20]; (2) Hamilton Anxiety Scale (HAMA) and Hamilton Depression Scale (HAMD) scores < 7; (3) right‐handed individuals; (4) age between 18 and 30 years; and (5) education level of undergraduate or graduate. The exclusion criteria were as follows: (1) with a history of epilepsy or confirmed epilepsy diagnosis; (2) a history of traumatic brain injury, severe physical illnesses, psychiatric disorders, or any other neuropsychiatric disorders that may interfere with the study, as well as related family history; (3) with metal implants, including non‐removable dentures; and (4) skin surface damage at the stimulation site. All participants underwent magnetic resonance imaging (MRI) using a Siemens 3.0 T Verio scanner (Siemens, Germany) with an eight‐channel radiofrequency coil. This step enabled the exclusion of organic disease and facilitated neuronavigation. This study was approved by the Human Research Ethics Committee of Nanjing Brain Hospital Affiliated to Nanjing Medical University (No: 2022‐KY150‐01). All participants provided written informed consent before the experiment.

### 2.2 Experimental Procedure

**FIGURE 1:**
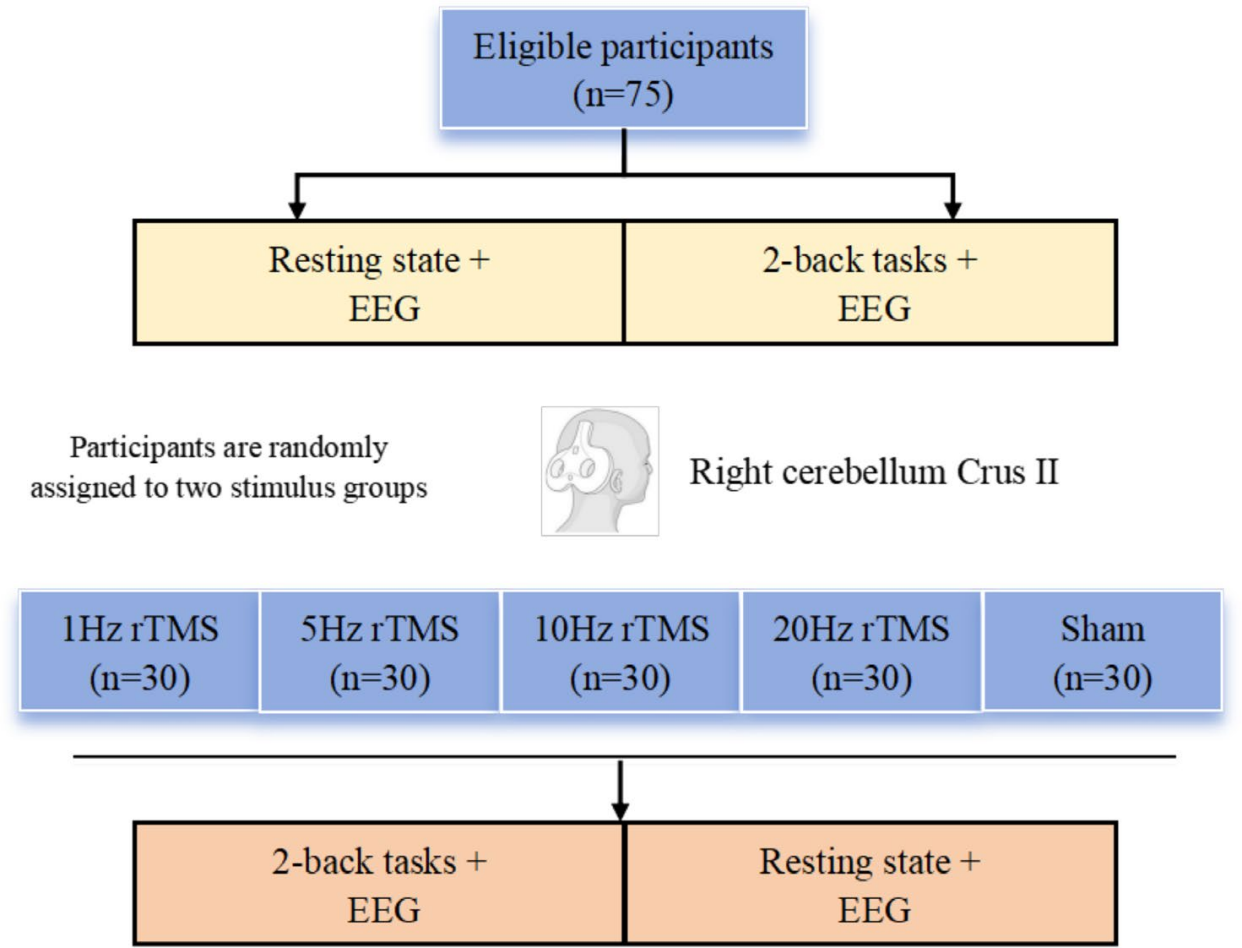
Experimental flow chart. Each subject was required to complete three modules of tasks. The first module was 10 min of resting state EEG acquisition and 15 min of 2‐back task EEG acquisition. After 3 min of rest, TMS intervention (1 Hz, 5 Hz, 10 Hz, 20 Hz, and sham stimulation) was randomly performed for about 20 min. After a rest of about 3 min, a 2‐back task and resting‐state EEG stimulation were performed.

The experimental procedure is shown in Figure 1. Five TMS conditions were used: 1 Hz rTMS (rTMS_1_), 5 Hz (rTMS_5_), 10 Hz (rTMS_10_), 20 Hz (rTMS_20_), and sham rTMS (rTMS_sh_). Each participant was randomly assigned to two conditions, with at least a 10‐day interval between sessions to avoid memory‐related or continuation effects of TMS [18]. Before the experiment, each participant practiced the N‐back task for 3–5 min to understand the procedure. For the experiment, electroencephalogram (EEG) data were recorded for 5 min at rest and during a 2‐back task, both before and after stimulation. The participants kept their eyes closed.

### 2.3 2‐Back Task

**FIGURE 2:**
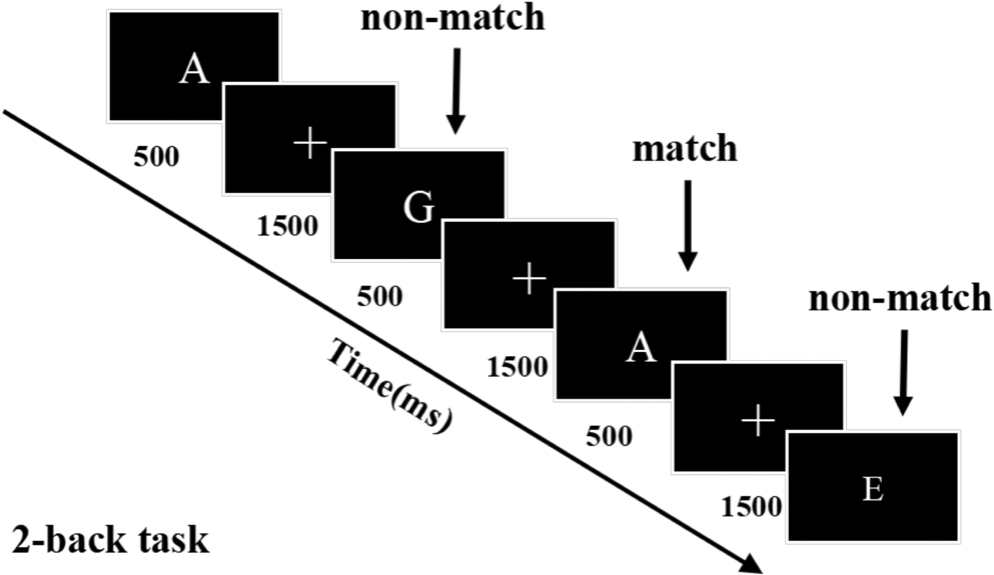
Schematic of the 2‐back task, illustrating the matching experiment for the 2‐back task. Letters were presented for 500 ms with 1500 ms intervals.

The 2‐back task is illustrated in Figure 2. It was programmed in MATLAB and using the Psychophysics toolbox to encode the order and time interval for presenting the visual stimulus on a computer screen [21]. The task lasted approximately 10 min. White letters from A to J were presented for 500 ms every 2000 ms; participants were instructed to press the “F” key if the current letter matched the one shown two steps earlier (2‐back) or the “J” key if the letters were inconsistent. Each task comprised 150 trials with 30% targets. Working memory performance was assessed using d' scores and reaction times for accurate responses. The d' score quantifies performance in terms of correct and incorrect hits as follows: d' score = Z (correct rate) − Z (error rate) [22].

### 2.4 TMS

rTMS was applied using a Magventure Rapid stimulator connected to a 70 mm figure‐eight coil (cool‐B65; MagPro X100, Denmark) with a maximum output of 6 T. The resting motor threshold (rMT) was obtained from the left motor cortex. Motor evoked potentials (MEPs) were recorded from the first dorsal interosseous muscle via Ag/AgCl electromyography electrodes. rMT was determined as the minimum intensity required to yield at least five MEPs > 0.05 mV. During stimulation, participants sat comfortably in a chair equipped with a headrest to stabilize their head. Using an individual MRI template for each participant, the algorithm created a virtual MRI that fit the shape of the participant's head. The MRI image was converted into Talairach space coordinates (X, Y, Z), and the position of the coil on the target area was precisely located using the neuronavigative system. The stimulation target was crus II of the right cerebellum at 32, −69, −40 coordinates.

Participants received 1800 pulses at the selected stimulation frequency (1 Hz, 5 Hz, 10 Hz, 20 Hz, and sham). Each participant was randomly assigned to two frequency band groups, resulting in 30 participants per frequency group. The stimulation intensity was set at 80% rMT, which was measured at each session. Sham stimulation was conducted using the same device with a specially designed coil that produces the noise of real rTMS without any electromagnetic energy.

### 2.5 EEG Recording and Data Preprocessing

Detailed procedures for EEG data recording and preprocessing are provided in the Appendix S1. Briefly, EEG recording was conducted using a 69‐ +2 channel EEG cap (Boricom Technology (Changzhou) Co. LTD.) [23]. The CPZ electrode was used as the reference, with FPZ serving as the ground. Data were sampled at a frequency of 1000 Hz, and electrode impedance was consistently maintained below 20 kΩ.

### 2.6 Event‐Related Potential Analysis

MATLAB and the Fieldtrip toolbox were used for event‐related potential (ERP) analysis [24]. Four ERPs were used: (N100 75–125 ms), (P150 130–190 ms), (N200 210–290 ms), and (P300 280–380 ms) [25, 26]. ERP comparisons were conducted at the whole‐cortex level using a cluster‐based permutation test to compare changes before and after stimulations. Waveforms were plotted using the mean of three prefrontal central electrodes (FC1, FCz, and FC2), which are less affected by electromyographic and electrophthalmogram artifacts.

### 2.7 Time‐Frequency Analysis

Using the Fieldtrip toolkit, ERPs were converted to the frequency domain using the “sliding Hanning window method” to measure event‐related oscillations during the 2‐back task, both before and after TMS. A temporal resolution of 10 ms per trial was used for each electrode, in 0.5 Hz steps from 3 to 80 Hz. The oscillatory power was averaged to calculate the total power of the activity. The baseline was defined as −200 ms to 0 ms, and the baseline type was “dB.” Normalized power oscillations were obtained by dividing all powers by the mean baseline value and considering the log change. Power values were averaged across the frequency bands of interest (theta (4–8 Hz), alpha (8–13 Hz), beta1 (13–20 Hz), beta2 (20–30 Hz), and gamma (30–80 Hz)). A cluster‐based permutation test was conducted for statistical comparisons.

### 2.8 PLV Network and Graph Theory Analysis

Brain network adjacency matrices were constructed for different frequency bands using phase‐locking value (PLV) (Appendix S1). First, average PLV values were compared before and after each stimulus condition. A threshold based on the top 30% of connection strengths was applied to generate sparse brain network matrices [27]. Finally, the Brain Connectivity Toolbox (BCT) (http://www.nitrc.org/projects/bct) was used to compute small‐world properties, including the clustering coefficient (Cp), shortest path length (Lp), and global efficiency (Eglob), for the binary brain networks.

### 2.9 Statistical Analysis

Statistical analyses were conducted using IBM SPSS 26.0 software (SPSS Inc., Chicago, Illinois, USA) and MATLAB. All data were analyzed using the Shapiro–Wilk test for normality. Normally distributed data were analyzed using parametric tests, whereas non‐normally distributed data were analyzed using non‐parametric tests. Chi‐square tests analyzed demographic and neuropsychological scale data. Between‐group comparisons were conducted using one‐way analysis of variance (ANOVA) for normally distributed data and the Kruskal–Wallis test for non‐normally distributed data. Electrophysiological data underwent cluster‐based permutation statistical analysis, which provides multiple comparisons that control for space (EEG electrodes) and time. First, changes before and after the intervention within each group were compared. Next, inter‐condition comparisons were performed using double impairments (post‐stimulus—pre‐stimulus). Monte Carlo *p*‐values were calculated from 5000 random permutations. To determine the cluster‐based statistical significance of two or more adjacent electrodes in all analyses, *p* < 0.05 was used as the threshold to control for multiple comparisons in space and time (*p* < 0.025, two‐tailed test). Inter‐group analysis was conducted using one‐way ANOVA, which included the pre‐stimulation, post‐stimulation, and the change Δ (post‐stimulation minus pre‐stimulation) comparisons. The false discovery rate (FDR) method was applied to correct for multiple comparisons.

For brain network parameters, paired‐sample two‐tailed *t*‐tests or Wilcoxon signed‐rank tests calculated the changes in graph theory parameters before and after stimulation for each group. The FDR‐corrected *p‐*values evaluated the changes in graph theory parameters in different groups.

For behavioral parameters, including d score and reaction time, one‐way analysis of variance (ANOVA) compared pre‐stimulus, post‐stimulus, double‐decrease (post‐stimulus minus pre‐stimulus), and change index X data. The Bonferroni method corrected for multiple comparisons. Pearson's correlation analysis assessed the correlation between 2‐back task‐related electrophysiology data and working memory performance:

$$X=\frac{\text{post stimulus}\left(\text{Post}\right)-\mathrm{pre}\;\text{stimulus}\left(\mathrm{Pre}\right)\;}{\mathrm{pre}\;\text{stimulus}\left(\mathrm{Pre}\right)}$$

## 3 Results

### 3.1 General Information

Table 1 lists the demographic and neuropsychological scale assessment results for the rTMS_1_, rTMS_5_, rTMS_10_, rTMS_20_, and rTMS_sh_ groups. No significant differences were observed among these groups in terms of education level and age. Additionally, no significant differences were observed regarding the MMSE and MOCA neuropsychological scores.

**TABLE 1:** General information.

	1 Hz rTMS	5 Hz rTMS	10 Hz rTMS	20 Hz rTMS	Sham rTMS	*p*
Age (SD)	24.560 (1.446)	24.100 (1.322)	24.769 (1.657)	24.464 (0.999)	24.808 (1.357)	0.237
Years of education (SD)	17.680 (0.802)	17.533 (0.9)	17.654 (1.325)	17.714 (0.854)	17.692 (0.928)	0.837
MMSE (SD)	29.960 (0.200)	29.833 (0.379)	29.885 (0.431)	29.929 (0.262)	29.808 (0.491)	0.477
MOCA (SD)	28.8 (1.118)	28.167 (1.440)	28.731 (1.313)	28.571 (1.260)	28.462 (1.174)	0.451
HAMA (SD)	2.080 (1.631)	1.767 (1.654)	1.538 (1.606)	1.857 (1.580)	1.192 (1.357)	0.249
HAMD (SD)	1.280 (0.98)	1.367 (1.273)	1.346 (1.441)	1.607 (1.370)	1.192 (1.470)	0.644

Abbreviations: HAMA, Hamilton Anxiety Scale; HAMD, Hamilton Depression Scale; MMSE, Mini‐Mental State Examination; MoCA, Montreal Cognitive Assessment.

### 3.2 TMS Safety and Tolerability

All participants showed good tolerance to TMS stimulation, and no serious adverse events directly related to the stimulation were observed. Five participants (3.7%) reported mild headaches after the stimulation, whereas one participant (0.7%) experienced transient nausea. These symptoms were mild and self‐limiting, resolving on their own 2 to 4 h later. No other subjective discomforts or abnormal physiological indicators were observed.

### 3.3 Behavioral Performance

To assess participants' response time during the 2‐back task, paired t‐tests were conducted within each stimulus set. The response time was significantly shorter after the rTMS intervention in all conditions. To account for a possible ceiling effect, a separate one‐way ANOVA was conducted on the pre‐stimulus data, post‐stimulus data, double‐minus data, and the change index X. As shown in Table 2, a group difference was observed in the post‐stimulation condition [*F* (4,130) = 2.538, *p* = 0.043]; however, this difference was statistically insignificant after applying Bonferroni correction. The change index X differed significantly between groups [*F* (4,130) =2.907, *p* = 0.024]. After Bonferroni correction, the rTMS_5_ group showed a significantly shorter change in response time than the rTMS_sh_ group (*p* = 0.028). No significant differences were observed between other groups.

**TABLE 2:** Reaction times in each group under the 2‐back task.

Reaction time (SD)	1 Hz rTMS	5 Hz rTMS	10 Hz rTMS	20 Hz rTMS	Sham rTMS	*p*	Adjusted *p*‐value
Pre	0.793 (0.163)	0.778 (0.124)	0.844 (0.177)	0.807 (0.145)	0.812 (0.195)	0.630	
Post	0.687 (0.127)	0.666 (0.125)	0.773 (0.177)	0.714 (0.137)	0.761 (0.182)	0.043	
Post − Pre	0.106 (0.099)	0.112 (0.099)	0.072 (0.066)	0.093 (0.079)	0.051 (0.075)	0.056	
$\frac{\text{Post}-\mathrm{Pre}}{\mathrm{Pre}}$	0.125 (0.100)	0.138 (0.122)	0.085 (0.079)	0.111 (0.090)	0.059 (0.085)	0.024	0.164^a^ 0.028^b^ 1.000^c^ 0.479^d^

Abbreviations: Post, Post‐stimulation; Pre, Pre‐stimulation; Sham, Sham stimulation.

^a^ 1 Hz versus Sham.

^b^ 5 Hz versus Sham.

^c^ 10 Hz versus Sham.

^d^ 20 Hz versus Sham.

### 3.4 ERP Analysis

**FIGURE 3:**
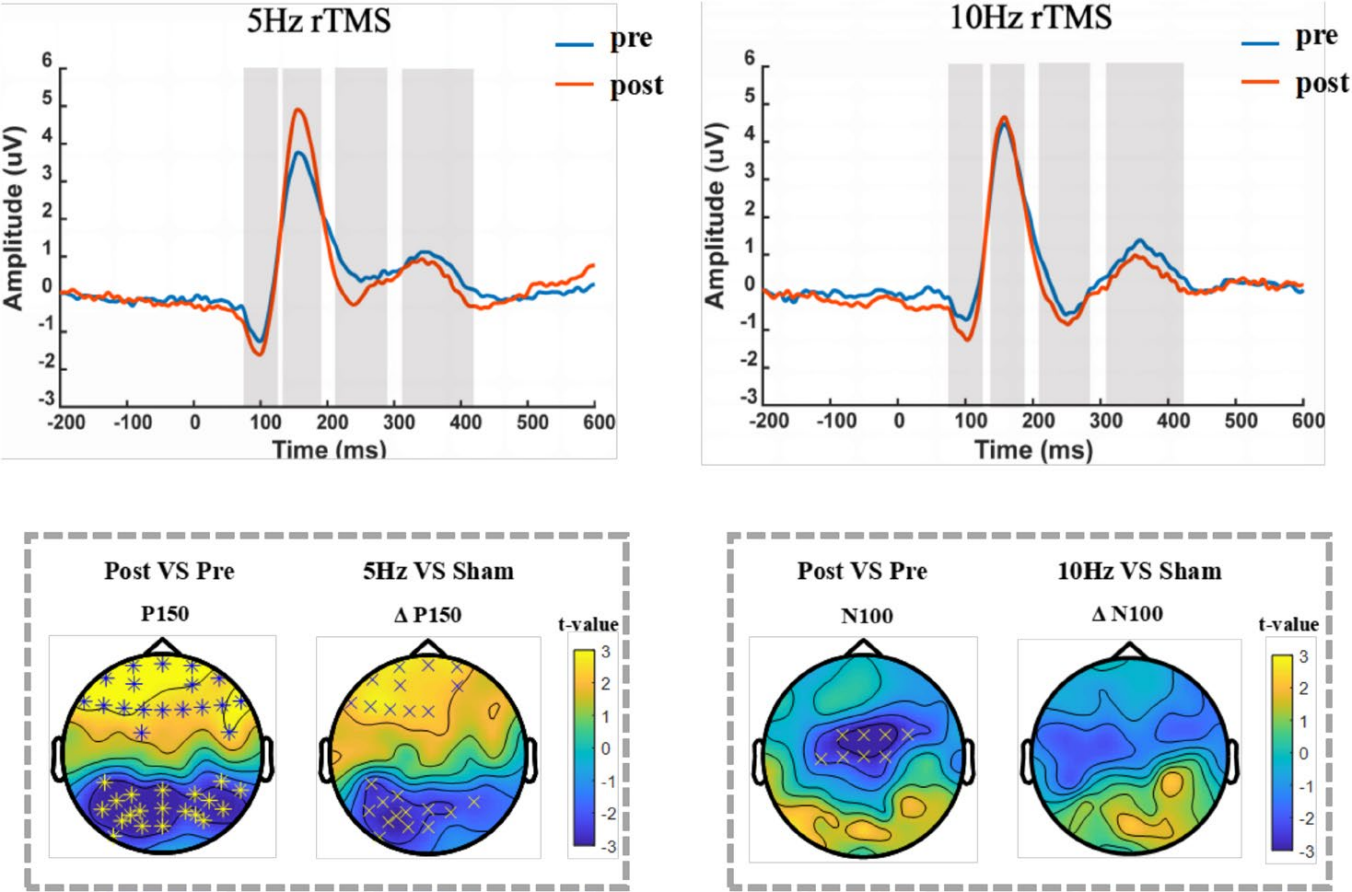
ERP analysis results for the average of three frontal‐central electrodes (FC1, FCz, and FC2) before and after stimulation in the rTMS_5_ group and rTMS_10_ group during the 2‐back task. Brain topographies represent *t* values before and after the intervention or for comparison between conditions. (Statistical significance is marked as *p < 0.01, ×*p* < 0.025). Post, Post‐stimulation; Pre, Pre‐stimulation; Sham, Sham stimulation.

During the 2‐back task, changes in the same participant groups were compared before and after stimulation. In the rTMS_5_ group, the P150 amplitude in the frontal and parietal regions increased significantly after stimulation (*p* = 0.0062 and *p* = 0.0026, respectively), whereas other peak amplitudes showed no significant differences. In the rTMS_10_ group, the N100 amplitude in the frontal central area increased significantly after stimulation (*p* = 0.011), whereas no other peak amplitudes showed significant differences (Figure 3). No significant changes were observed at various peaks in the remaining groups (Appendix S1).

**FIGURE 4:**
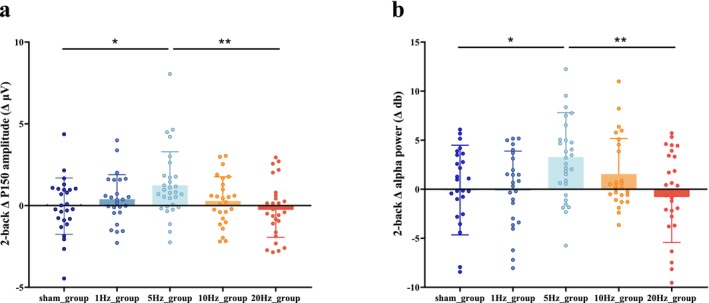
(a) Shows a dot plot of the differences in ΔP150 across different groups, with the rTMS_5_ group significantly higher than rTMS_sh_ and rTMS_20_ groups. (b) Shows a dot plot of the differences in ΔP150 frequency band across different groups, with the rTMS_5_ group also significantly higher than rTMS_sh_ and rTMS_20_ groups. Statistical significance is marked as **p = 0.01, *p < 0.05.

For comparisons across conditions, ERP amplitude changes induced by rTMS interventions at different frequencies were calculated by constructing different waveforms (post‐intervention—pre‐intervention). The difference values (Δ) between each stimulation condition were compared. Relative to the sham group, the rTMS_5_ group showed an increase in the amplitude of difference waves at ΔP150 in both the frontal and parietal regions (*p* = 0.023 and *p* = 0.016, respectively; Figure 3). No significant differences were observed in the peak amplitudes of other groups, compared with the sham group (Appendix S1). ANOVA yielded similar results, with a significant difference in ΔP150 between groups [*F* (4,130) = 3.196, *p* = 0.015]. After FDR correction, the ΔP150 in the rTMS_5_ group was higher than that in the rTMS_sh_ (*p* = 0.035) and rTMS_20_ (*p* = 0.01) groups (Figure 4a).

### 3.5 Time‐Frequency Analysis

**FIGURE 5:**
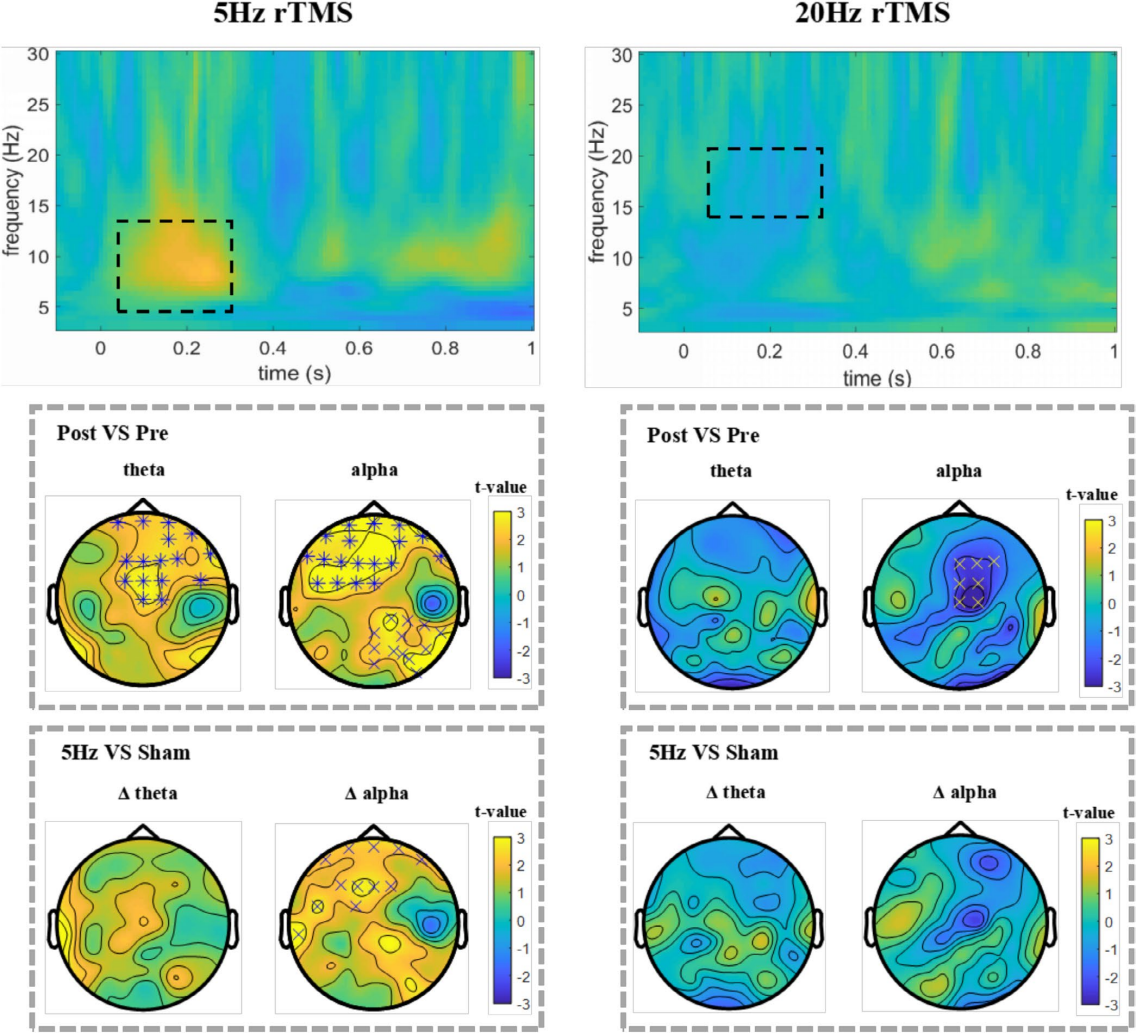
Time‐frequency analysis results of the double decrement values (post‐stimulation minus pre‐stimulation) in the rTMS5 group and rTMS10 group during the 2‐back task at the average of three frontal‐central electrodes (FC1, FCz, and FC2). Brain topographies represent *t* values before and after the intervention or for comparison between conditions. (Statistical significance is marked as *p < 0.01, ×*p* < 0.025).

To evaluate the regulatory effects of rTMS on oscillatory activity during a working memory task, a time range from 50 to 350 ms was selected. Theta, alpha, beta1, beta2, and gamma oscillatory activities were compared under different stimulation conditions. Figure 5 presents the cluster‐based permutation tests comparing pre‐ and post‐stimulation changes within each group. Relative to pre‐intervention levels, the rTMS_5_ intervention significantly increased the theta band power in the frontal region (*p* = 0.005). The post‐intervention power in the alpha band in the frontal and right parietal regions also significantly increased (*p* = 0.0036 and 0.011, respectively). A trend toward enhanced power in the beta1 band was observed in the central region (*p* = 0.030), but no significant differences were observed in other frequency bands. Hence, rTMS_5_ can significantly increase theta and alpha oscillations and may affect the beta1 band. In the 20 Hz rTMS group, the post‐intervention power in the beta1 band in the right central region significantly decreased (*p* = 0.019). No significant differences for any frequency band were observed in the rTMS_1_, rTMS_10_, or rTMS_sh_ groups (Appendix S1). Next, changes in Δ values (post‐intervention—pre‐intervention) were compared among different groups using cluster‐based permutation tests. Compared with the sham group, the rTMS_5_ group exhibited increased power in the Δ alpha band in the frontal region (*p* = 0.011), but no significant differences in other frequency bands. The ANOVA yielded similar results, with a significant difference in Δalpha between groups [*F*(4,130) = 3.476, *p* = 0.010]. After FDR correction, the Δalpha in the rTMS_5_ group was significantly higher than that in the rTMS_sh_ (*p* = 0.043) and rTMS_20_ (*p* = 0.01) groups (Figure 4b).

### 3.6 Brain Network Analysis

**FIGURE 6:**
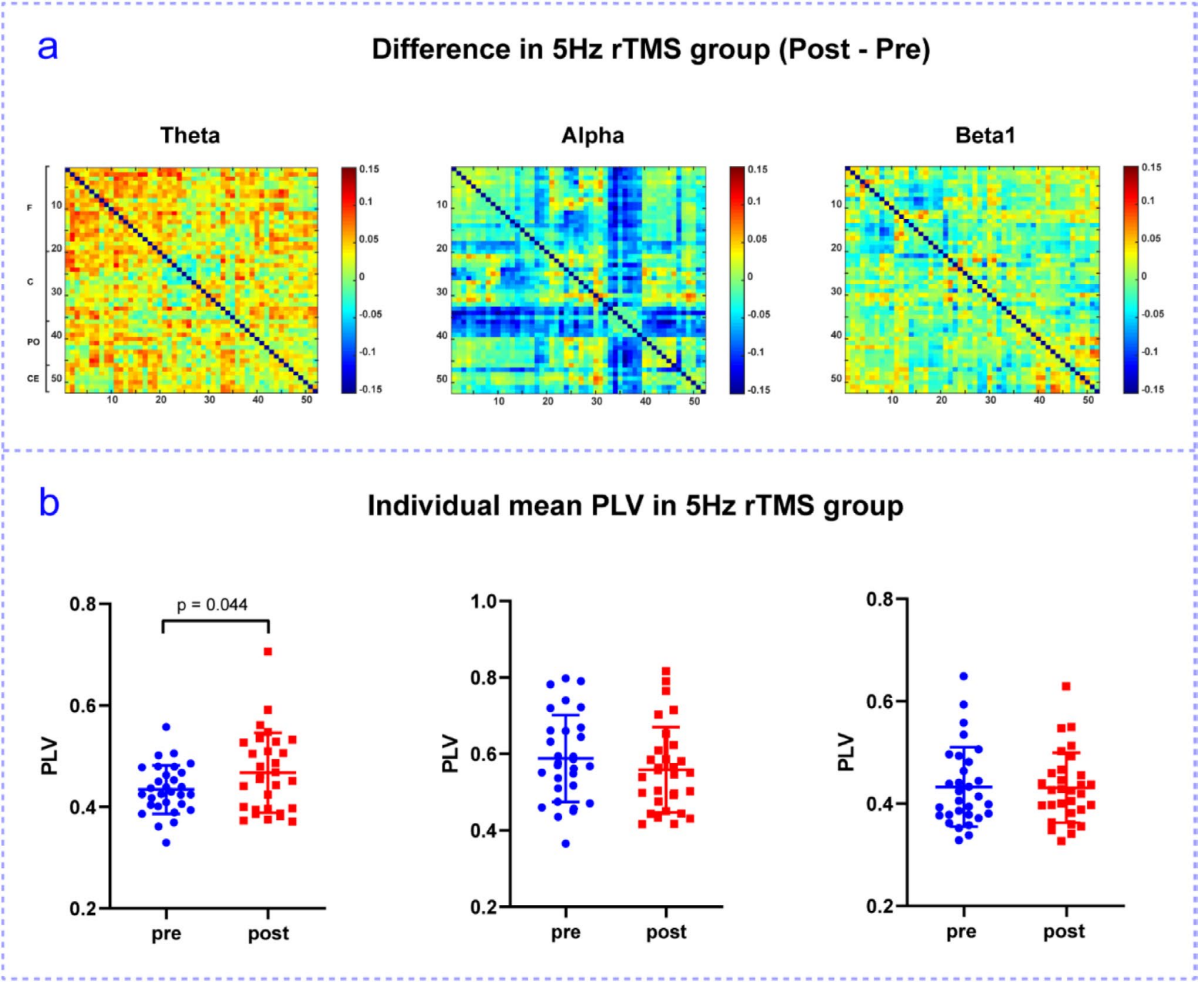
Matrix plots of phase‐locked values (PLV) and difference plots of mean PLV in the θ, α, and β1 bands between the rTMS_5_ group. (a) represent the difference in the mean PLVs in the fully connected matrix within the group for the rTMS_5_ group (post‐stimulus–pre‐stimulus), respectively. (b) represent scatter plots of the mean PLVS for individual participants in the rTMS_5_ group. Color bars depict the difference in mean PLV after stimulation compared with before stimulation.

The mean PLV of each frequency band was calculated before and after the rTMS interventions. Compared with pre‐stimulation, the rTMS_5_ group exhibited an increasing trend in phase synchronization in the θ frequency band, a decreasing trend in the α frequency band, and a moderate change in the β frequency band (Figure 6a). In the θ frequency band, PLV values were significantly higher after the rTMS_5_ intervention than before the intervention (Figure 6b). Furthermore, no significant differences were observed in the 1 Hz, 10 Hz, 20 Hz, or sham stimulation groups (Appendix S1).

**FIGURE 7:**
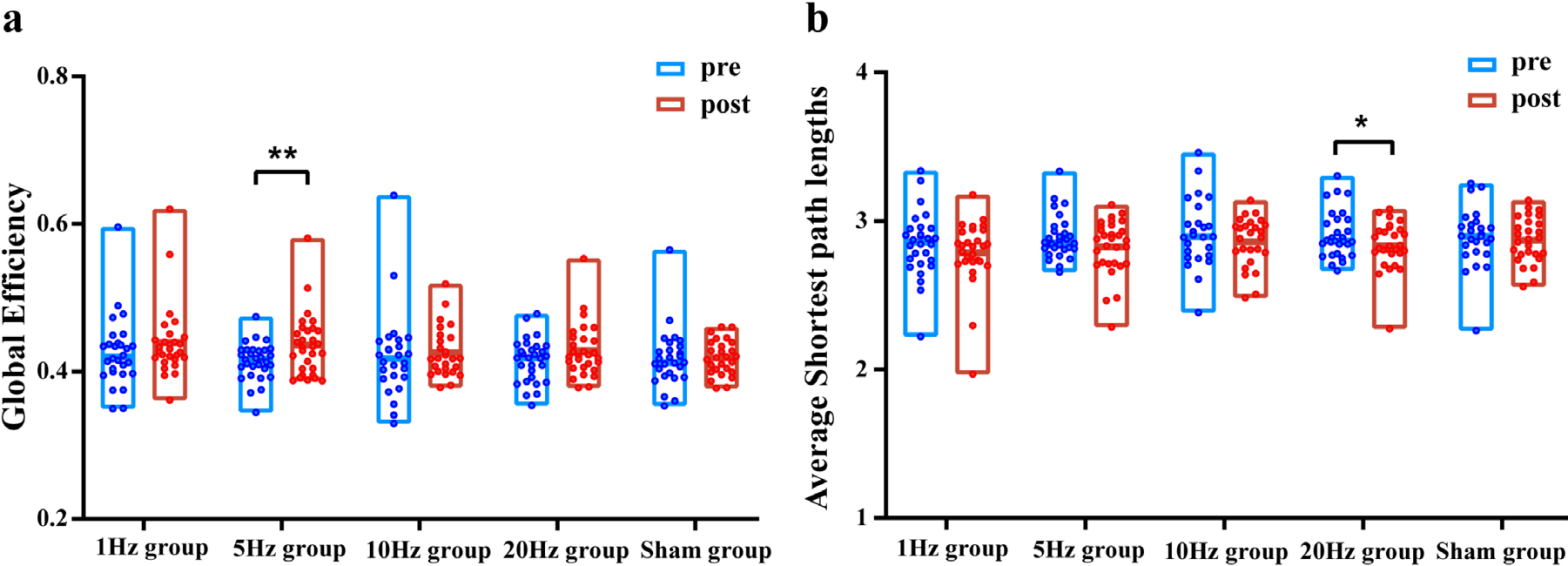
Box plot of global efficiency and shortest path length for each group in the θ band. (a) represents global efficiency, and (b) represents the shortest path length. Blue represents pre‐stimulation, red represents post‐stimulation. Statistical significance is marked as **p = 0.01, *p < 0.05.

Next, parameters, including the average Cp, Eglob, average characteristic Lp, and local efficiency of the PLV undirected matrix, were analyzed using graph theory. The 5 Hz group did not show any difference in the average Cp or characteristic Lp; however, a significant difference in Eglob was observed in the theta band (*p* = 0.006) (Figure 7). In the rTMS_20_ group, no significant difference in the average clustering coefficient or Eglob was observed in each frequency band; however, the characteristic Lp was significantly reduced in the theta frequency band (*p* = 0.043). No significant differences were observed in these indices for the rTMS_1_, rTMS_10_, or rTMS_sh_ groups. The rTMS_5_ group showed significantly enhanced local efficiency in the prefrontal lobe, left central region, parieto‐occipital lobe, and cerebellum in the θ frequency band, but no significant differences in other frequency bands (Appendix S1).

### 3.7 Correlation Analysis

**FIGURE 8:**
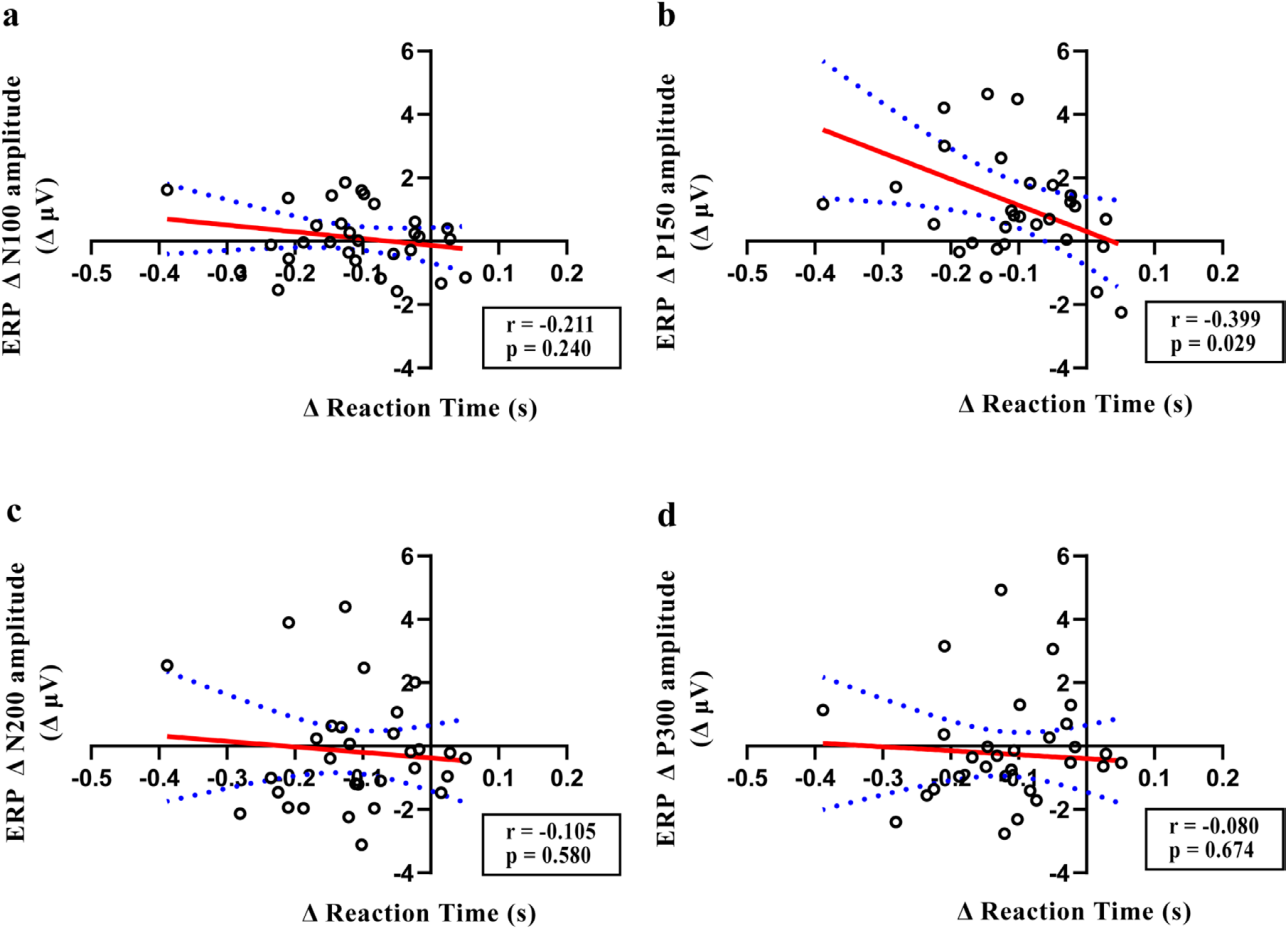
Correlation between the average amplitude of each ERP (N100, P150, N200, P300) and reaction time in the rTMS_5_ group during the 2‐back task. (a–d represent correlations for ΔN100, ΔP150, ΔN200, and ΔP300, respectively).

**FIGURE 9:**
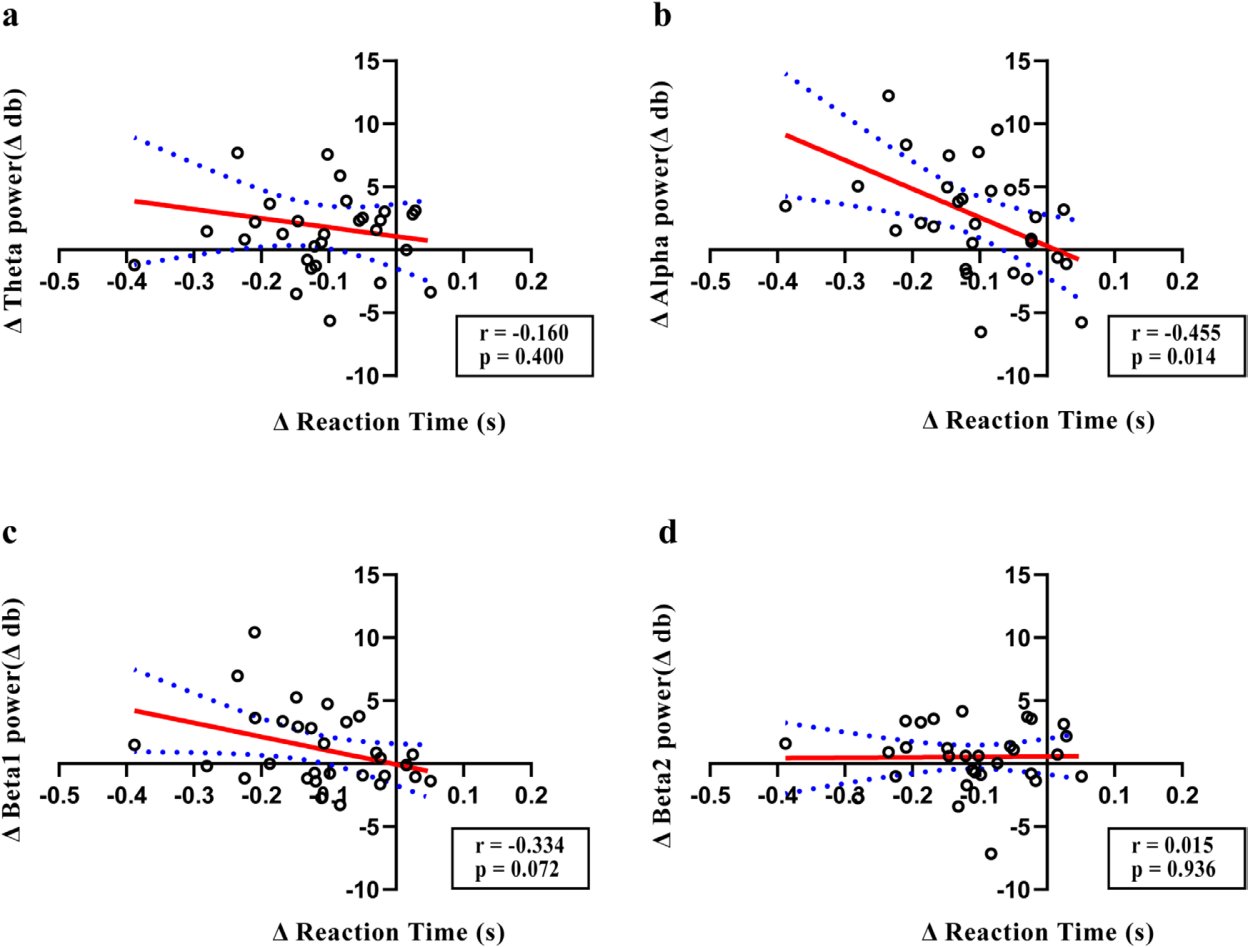
Correlation between oscillatory activity and reaction time in each frequency band in the rTMS group during the 2‐back task. (a–d represent correlations for ΔTheta, ΔAlpha, ΔBeta1, and ΔBeta2, respectively).

To investigate the association among behavioral performance, ERP data, and oscillatory activity, correlation analysis was conducted using the Δ values. A significant negative correlation was observed between ΔP150 and Δreaction time in the rTMS_5_ group (*r* = −0.399, *p* = 0.029), but not in other peaks (Figure 8b). No correlation was observed between ΔP150 and Δreaction time in other groups. Δalpha power was negatively correlated with the Δreaction time (*r* = −0.455, *p* = 0.014), although no significant correlations were observed in other frequency bands (Figure 9b). No significant correlations were observed in other groups.

To further explore the relationship between ERP and oscillatory activity, a correlation analysis was conducted using differential amplitude and differential power (Δ). The Δ P150 was positively correlated with the Δ theta (*r* = 0.557, *p* = 0.001), Δ alpha (*r* = 0.679, *p* = 0.000), and Δ beta1 (*r* = 0.544, *p* = 0.002) frequency bands in the rTMS_5_ group. Similar patterns were observed for other groups, except for the TMS_1_ group (Appendix S1).

## 4 Discussion

The study investigated the effects of applying different frequencies of rTMS stimulation to the right cerebellar Crus II area on neurophysiological responses and working memory. All rTMS interventions improved working memory compared with sham stimulation, with rTMS_5_ producing the most pronounced effect. rTMS_5_ significantly enhanced the P150 amplitude and elicited stronger θ and α‐band oscillatory activity than the sham condition. Analysis of brain network topology demonstrated that rTMS_5_ increased global efficiency in the theta band across brain networks and enhanced local efficiency in various brain regions. Correlations were observed between neurophysiological data and behavioral outcomes. Specifically, the P150 amplitude and α power changes in the rTMS_5_ were closely associated with reaction times. Overall, applying rTMS_5_ to the right cerebellar Crus II area effectively enhances working memory in healthy adults and improves overall brain network connectivity and efficiency at rest.

### 4.1 Effects of rTMS on Working Memory

For the 2‐back task, working memory performance improved over time regardless of the stimulus condition. This improvement may be attributed to a training effect. Behavioral performance for the 2‐back tasks is enhanced after high‐frequency rTMS or iTBS interventions in healthy people [28, 29, 30, 31]. A meta‐analysis found that cerebellar TMS successfully modulates cognitive performance, affects accuracy and reaction time, and exerts robust cumulative effects [15]. Yao et al. reported that rTMS_5_ to the cerebellar crus II region enhanced general cognitive performance and memory scores in individuals with mild AD. Although a study examining the effects of right hemisphere TMS on verbal working memory reported a significant increase in error rates following the intervention, it employed a combination of online TMS and paired 20 Hz stimulation [32]. Another study found that TBS treatment on both sides of the cerebellum led to only modest improvements in n‐back performance, while cTBS inhibited verbal working memory [33]. These findings suggest that different stimulation parameters can have distinct effects on working memory. Consistent with our results, 5 Hz rTMS appears to have a more pronounced impact on cognitive improvement.

### 4.2 Effects of rTMS on ERPs During the 2‐Back Task

P150 is an early component of ERPs (also termed P2); it is related to initial stimuli processing and the allocation of attention resources, reflecting the early stage of cognitive processing [34, 35, 36]. During the 2‐back task, a significant increase in P150 amplitude was observed after rTMS_5_, primarily in the prefrontal and parietal‐occipital regions. These changes were significant compared with the rTMS_sh_ condition, and the affected regions were consistent with those associated with working memory [37]. In a previous study, an increase in functional connectivity between the cerebellar crus II and prefrontal cortex was observed after rTMS_5_ [14]. Hence, rTMS_5_ over the crus II region may modulate working memory by impacting memory processing in the prefrontal and parietal regions. In contrast, rTMS_10_ only resulted in significant differences before and after the intervention, without significant differences compared with rTMS_sh_.

### 4.3 Effects of rTMS Stimulation on Neural Oscillatory Activity

To date, most TMS research has focused on the cerebral cortex, with limited attention to TMS‐induced oscillatory changes in the cerebellum. In the present study, the cerebellar rTMS_5_ caused θ oscillations and spread to the α and possibly β1 frequency bands. Hence, rTMS_5_ can influence working memory by generating larger and broader neural oscillation activity. θ oscillations play an important role in cognitive processes and are associated with maintaining information during working memory tasks [38, 39] and memory‐related networks [40]. θ band TMS targeting the crus I/II area alters resting‐state fMRI connectivity between and within the cerebellar and hippocampal networks [41]. In the present study, between‐group analysis suggested that the rTMS_5_ group showed a significant increase in α power, compared with other groups. α‐oscillatory activity has been strongly associated with cognitive function [42, 43], and α power may change after TMS [44], consistent with the present findings. Thus, cerebellar TMS may directly interact with underlying brain oscillations via TMS‐induced entrainment, enhancing brain oscillatory activity and affecting working memory performance.

### 4.4 Effects of rTMS on Resting‐State Brain Networks

The topological parameters of brain networks have been strongly associated with cognitive functions, such as working memory and emotion processing [45, 46]. TMS modulates changes in brain functional connectivity [47]. In this study, 5 Hz rTMS over the cerebellar crus II significantly enhanced global efficiency in the θ frequency band, augmenting the capacity to process and transmit information. Furthermore, the θ band activity during the resting state may reflect the brain's preparation and regulatory state for upcoming cognitive tasks [38]. Cerebellar rTMS may influence working memory by enhancing frontal lobe activity and increasing intra‐frontal lobe functional connectivity. Additionally, the shortest path length decreased after stimulation in the 20 Hz rTMS group, which might be attributed to resource redistribution by shortening information transmission pathways to reduce information consumption. This effect may be related to selecting the cerebellum as the target, necessitating further investigation in the future.

### 4.5 Relationship Analysis

The rTMS5 group demonstrated that increased P150 amplitude was associated with reduced reaction time during the 2‐back task. Increased alpha activity after rTMS5 was significantly correlated with shorter reaction times, suggesting that enhanced α oscillatory activity improves task performance. Increased alpha power reflects easier task performance and faster reaction times [42, 48], particularly in frontal and posterior regions during memory tasks [49]. Additionally, P150 was positively correlated with θ, α, and β1 oscillations; therefore, rTMS5 enhances ERPs and neural oscillatory activity, improving working memory. No correlation between θ power and behavior was observed, consistent with the finding that pseudo‐stimulation enhanced only α oscillatory activity. After the cerebellar 5 Hz rTMS intervention, θ oscillatory activity related to the frontal cortex was generated initially and subsequently spread to the frontal‐parietal lobe to generate greater α oscillatory activity, explaining the finding. However, the mentioned mechanisms warrant further exploration to validate these results.

To the best of knowledge, this is the first study to apply five commonly used frequency bands, including both low‐ and high‐frequency stimulation, to clarify the effects of cerebellar rTMS on cognitive function regulation. Notably, low‐frequency stimulation had minimal effects on the working memory task and resting state EEG results. Only rTMS5 had a significant effect on working memory and resting state, whereas the effects of other frequency bands were relatively limited. rTMS5 may induce θ oscillations in the hippocampal frontal cortex and α oscillations in the parietal cortex associated with working memory through a resonance effect. This is because crawling fibers in the cerebellar Purkinje and deep nuclei cells transmit periodic signals of theta frequencies associated with working memory and coordinate control of the corticocerebellar circuit through low‐frequency oscillations [50].

### 4.6 Limitations

This study has several limitations. First, because of the relatively short duration of the TMS intervention, cerebellar neural modulation effects might not have been entirely activated. Second, the high variability of EEG signals across individuals limited the effectiveness of the EEG‐based topological brain network information analysis. Additionally, the sample was restricted to healthy young adults with high cognitive levels, which might have introduced ceiling effects in the overall accuracy of the dual‐back task, limiting the generalizability of the results. Future research is warranted to further explore three areas: (i) extending the duration of cerebellar TMS intervention to observe cumulative effects, (ii) using multimodal techniques (such as combining TMS‐EEG with synchronize cortical excitability dynamics) to analyze neural regulation mechanisms, and (iii) expanding sample diversity to examine the differential effects of the intervention across different cognitive groups. Furthermore, by comparing the effects of stimulation protocols (such as cTBS and iTBS), strategies to enhance the working memory of cerebellar TMS intervention paradigms can be further optimized.

## 5 Conclusions

Overall, applying rTMS_5_ over the crus II region of the right cerebellum effectively improves electrophysiological activity and behavioral performance in the resting state and during a working memory task. These findings underscore the importance of cerebellar rTMS_5_ in regulating cognition and provide valuable insights for its clinical applications.

## Author Contributions

Bo Song: conceptualization, data curation, investigation, methodology, software, validation, visualization, writing – original draft, writing – review and editing. Minjie Tian: conceptualization, investigation, supervision, writing – review and editing. Tong Wang: data curation, investigation, writing – review and editing. Xixi Wang: data curation, investigation, writing – review and editing. Xing Ye: methodology, software, writing – review and editing. Qun Yao: conceptualization, supervision, writing – review and editing. Jingping Shi: conceptualization, supervision, project administration, funding acquisition, writing – review and editing. Kuiying Yin: conceptualization, supervision, funding acquisition, writing – review and editing.

## Conflicts of Interest

The authors declare no conflicts of interest.

## Supporting information


Appendix S1.

## Data Availability

The data that support the findings of this study are available on request from the corresponding author. The data are not publicly available due to privacy or ethical restrictions.
