# Supplementary material for: Effects of Cerebellar Repetitive Transcranial Magnetic Stimulation at Different Frequencies on Working Memory: An EEG Study
Source: CNS Neurosci Ther. 2025 Jul 16;31(7):e70491. doi: 10.1111/cns.70491 (PMC12264452; doi:10.1111/cns.70491)
Supplement: Supplementary file 1 — Appendix S1. [file CNS-31-e70491-s001.zip › Supplementary material_1.docx]

**Supplementary Material**

**1. EEG recording**

We used an EEG cap with 69 +2 channels (Boricom Technology (Changzhou) Co., LTD.). Electrode positions were placed according to the 10-20 international standard lead system with 69 fixed electrodes, 1 reference electrode at the standard CPz position, and 1 ground electrode at the standard AFz position. The EEG cap covers 59 conventional electrode sites in the whole brain area and is equipped with PO9, O9, IZ, O10, PO10, CB1, CBz, and CB2 electrodes in the cerebellum of the human body. All data were sampled at a frequency of 1000 Hz. Every electrode impedance was kept below 20 kΩ.

**2. EEG data preprocessing**

For EEG recording during the 2-back task, we utilized MATLAB and EEGLAB toolbox to preprocess the raw EEG data [1]. Firstly, we performed channel localization and removed useless electrodes. Secondly, a 48 to 52 Hz notch filter was applied to the data to remove the 50 Hz frequency interference in the power system, and a 0.1 Hz high-pass filter and an 80 Hz low-pass filter were applied to remove high-frequency non-EEG components. Thirdly, we segmented the whole data (-500ms, 1000ms) to retain all segments with correct responses. Baseline correction (-300ms, -50ms) was also performed to remove baseline drift. Fifthly, we removed the bad segments and bad channels and compensated the adjacent channels by the interpolation method. Sixthly, we performed whole-brain average weight reference. Finally, we performed independent component analysis on the data, drew the component map using EEGLAB, and manually removed the noisy components such as electrooculogram, electromyogram, and electrocardiogram.

1. **Construction of the PLV network**

Network analysis is a method based on considering nodes (vertices) and edges (connections), to apply network analysis in functional neuroimaging techniques, each brain region or recording electrode can be considered a node, and the correlation/synchrony between regions or electrodes is assumed to be an edge [2]. In this study, each EEG electrode (channel) is considered as a node, and the phase locking value (PLV) between EEG channels acts as an edge, which can be used to detect changes in the synchrony of neural activity represented by EEG signals.

Phase synchronization between two narrowband signals is often characterized by PLV. High phase-locking value (PLV) between two brain regions indicates high synchronization [3]. A 67×67 brain function network is constructed by calculating the phase locking value between two channels as the edge of the brain function network by the following formula. Given that the instantaneous phases of two signals x(t) and y(t) at time t are ϕx(t) and ϕy (t), PLV is defined as:

$${PLV}_{t}=\frac{1}{N}|\sum_{n=1}^{N} e^{i\Delta\phi(t)}|,\Delta\phi\left( t \right)=\phi_{x}\left( t \right)-\phi_{y}(t)$$

Where t represents the time point, N represents the number of sample points of the signal, and ∆ϕ(t) represents the phase difference between the two electrodes at time t [4]. We used PLV to construct adjacency matrices of brain networks in different frequency bands, to identify changes in brain networks before and after stimulation. After obtaining the brain network, we used the top 30% of the connection strength as the threshold to generate the sparse matrix of the brain network [5]. Next, we compared and analyzed the difference in connection synchronization across all groups in the binary brain network. The difference matrix was obtained by subtracting the binary adjacency matrix before each stimulation from the binary adjacency matrix after each stimulation. The difference matrix was used to construct the brain functional network, and the connection synchronization differences between different brain regions were analyzed.

**Results**

1. **Behavioral performance**

To assess participants’ d prime (d’) during the 2-back task, a paired t-test was performed within each stimulus set. The results revealed that the 5 Hz rTMS group (P = 0.013), 10 Hz rTMS group (P = 0.003) and the 20 Hz rTMS group (P = 0.016) demonstrated a significant increase in accuracy after stimulation, while the 1 Hz rTMS group and sham stimulation group showed no significant improvement (Table S1). Subsequently, we performed one-way ANOVA on the pre-stimulation, post-stimulation, dual reduction, and change index X separately. According to the results presented in Table S2, no significant differences were found in the pre-stimulation, post-stimulation, dual reduction, and change index X.

Table S1 d prime within each group under the 2-back task

| d’(SD) | Pre | Post | P_value |
| --- | --- | --- | --- |
| 1Hz Group | 3.30(1.00) | 3.48(1.12) | 0.228 |
| 5Hz Group | 3.19(1.09) | 3.65(0.89) | **0.013** |
| 10Hz Group | 3.28(0.83) | 3.62(0.84) | **0.003** |
| 20Hz Group | 3.17(1.02) | 3.52(0.90) | **0.016** |
| Sham Group | 3.22(0.81) | 3.47(0.71) | 0.056 |

*Significant changes from baseline highlighted in bold. Pre: Pre-stimulation, Post: Post-stimulation, Sham: Sham stimulation.

Table S2 d prime in each group under the 2-back task

| d’(SD) | 1Hz  Group | 5Hz  Group | 10Hz  Group | 20Hz  Group | Sham  Group | P_value |
| --- | --- | --- | --- | --- | --- | --- |
| Pre | 3.462  (0.779) | 3.245  (0.936) | 3.447  (0.952) | 3.313  (0.952) | 3.226  (0.863) | 0.810 |
| Post | 3.627  (1.060) | 3.619  (0.866) | 3.641  (0.929) | 3.682  (0.829) | 3.424  (0.861) | 0.861 |
| Post - Pre | 0.165  (0.811) | 0.374  (0.866) | 0.195  (0.509) | 0.370  (0.780) | 0.198  (0.504) | 0.690 |
| $\frac{Post - Pre}{\mathrm{Pre}}$ | 0.053  (0.254) | 0.174  (0.350) | 0.083  (0.212) | 0.166  (0.304) | 0.071  (0.182) | 0.320 |

Pre: Pre-stimulation, Post: Post-stimulation, Sham: Sham stimulation.

1. **ERP analysis**

No significant differences were found before and after the rTMS_1_, rTMS_20_, and rTMS_sh_ conditions at any time point (all p > 0.025) or peak values. Similarly, there were no significant differences in peak amplitudes among these groups compared to the sham stimulation group (Figure S1).
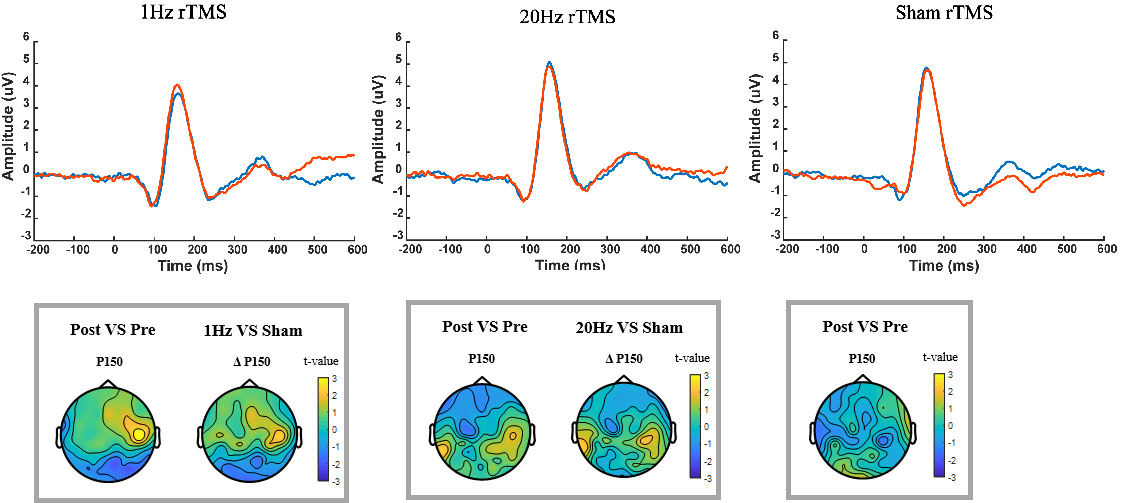


Fig S1. ERP analysis results for the average of three frontal-central electrodes (FC1, FCz, and FC2) before and after stimulation in the rTMS_1_ group, rTMS_20_ group and rTMS_sh_ group during the 2-back task. Brain topographies represent t values before and after the intervention or for comparison between conditions. (Statistical significance is marked as *p <.01, ×p <.025). Pre: Pre-stimulation, Post: Post-stimulation, Sham: Sham stimulation.

1. **Time-frequency analysis**

No significant differences were found before and after the rTMS_1_, rTMS_20_, and rTMS_sh_ conditions at any frequency band (all p > 0.025). Similarly, there were no significant differences in any frequency band among these groups compared to the sham stimulation group (Figure S2).


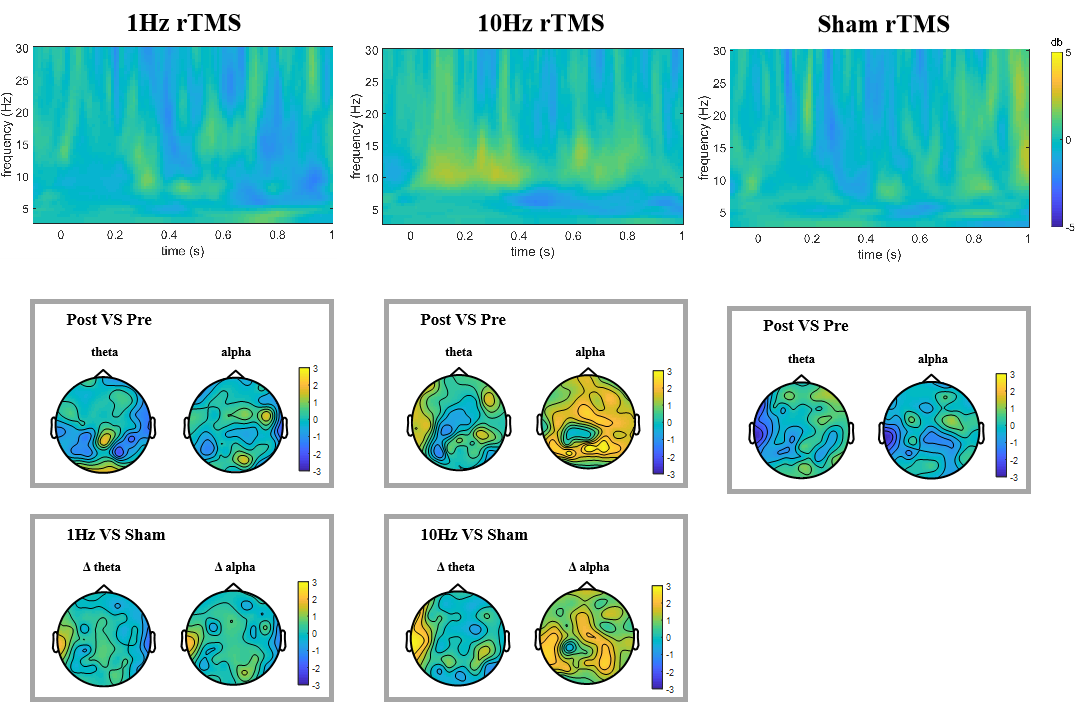


Fig.S2. Time-frequency analysis results of the double decrement values (post-stimulation minus pre-stimulation) in the rTMS1 group, rTMS10 group and rTMSsh group during the 2-back task at the average of three frontal-central electrodes (FC1, FCz, and FC2). Brain topographies represent t values before and after the intervention or for comparison between conditions. (Statistical significance is marked as *p <.01, ×p <.025). Pre: Pre-stimulation, Post: Post-stimulation, Sham: Sham stimulation.

1. **Brain network analysis**

No significant differences were found in the mean PLV before and after the rTMS1, rTMS10, rTMS20, and rTMSsh conditions at any frequency band (all p > 0.025) (Figure S3).


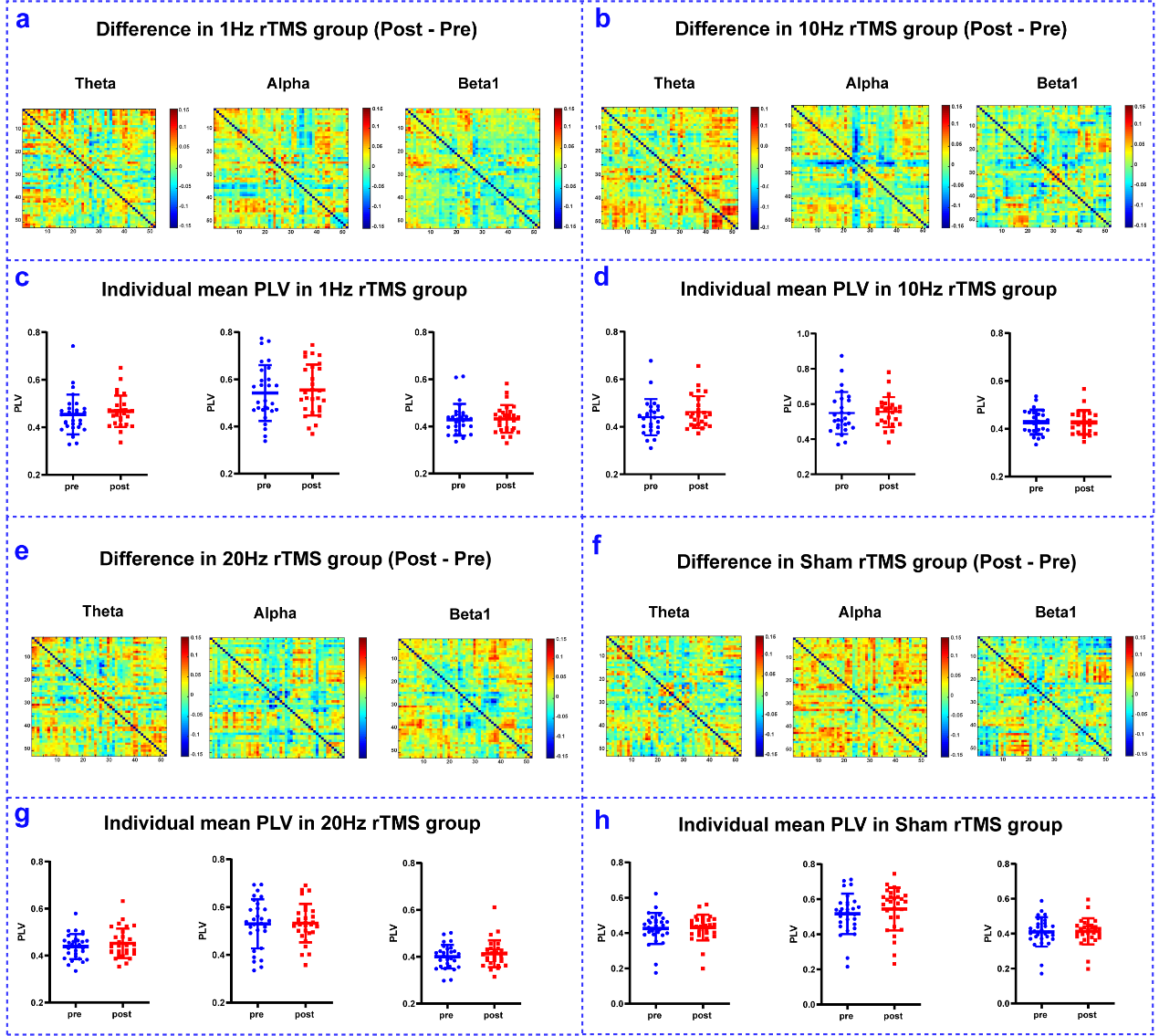


Fig S3. Matrix plots of PLV and difference plots of mean PLV in the θ, α and β1 bands between the rTMS1, rTMS10, rTMS20 and rTMSsh. Figures S3a, b, e, and f represent the differences in the mean PLVs in the fully connected matrix within the group for the rTMS1, rTMS10, rTMS20 and rTMSsh condition (Post-stimulus - Pre-stimulus), respectively. Figures S3c, d, g, and h represent scatter plots of the mean PLVs for individual participants in rTMS_1_, rTMS_10_, rTMS_20_, and rTMS_sh_, respectively. The color bars depict the difference in mean PLV after stimulation compared to before stimulation.

No significant differences in global efficiency and shortest path length were found among the groups in the delta, alpha, and beta1 bands (all p > 0.025). (Figure S5 – S7).


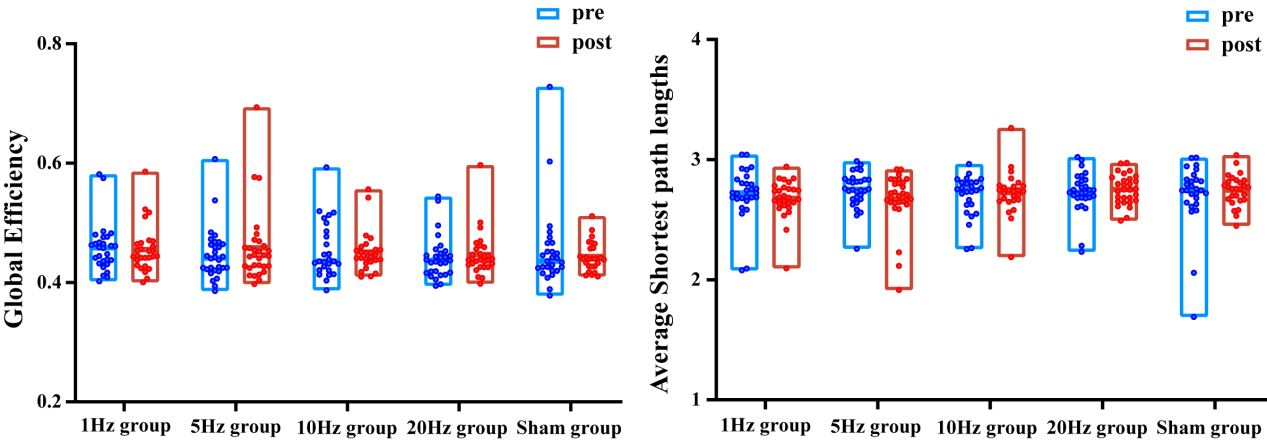


Figure S4. Violin plot of global efficiency and shortest path length for each group in the delta band. Fig S4 a. represents global efficiency, and Fig S4 b. represents the shortest path length. Blue represents pre-stimulation, red represents post-stimulation.


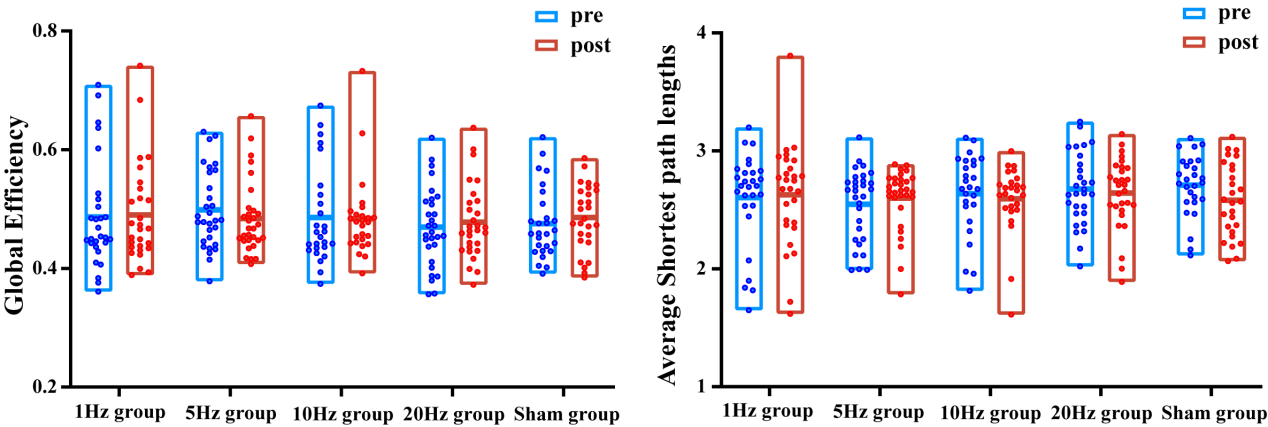


Figure S5. Violin plot of global efficiency and shortest path length for each group in the alpha band. Fig S5 a. represents global efficiency, and Fig S5 b. represents the shortest path length. Blue represents pre-stimulation, red represents post-stimulation.


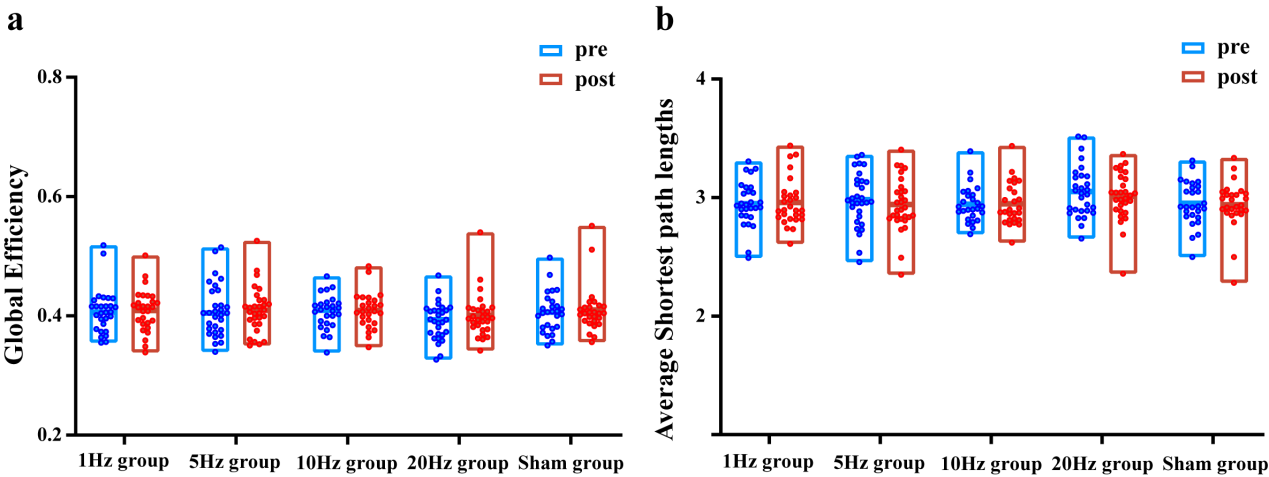


Figure S6. Violin plot of global efficiency and shortest path length for each group in the beta1 band. Fig S6 a. represents global efficiency, and Fig S6 b. represents the shortest path length. Blue represents pre-stimulation, red represents post-stimulation.

In the 5 Hz rTMS group, significant differences in theta band local efficiency were found in all brain regions except the right central area (Tables S4). No significant differences were found in local efficiency across the rTMS_1_, rTMS_10_, rTMS_20_ and rTMS_sh_ groups for the delta, alpha, theta and beta1 bands (all p > 0.025) (Tables S3–S6).

Table S3. Local efficiency of each brain region across groups in the delta frequency band before and after rTMS

| Delta band | Group 1Hz | | | Group 5Hz | | | Group 10Hz | | | Group 20Hz | | | Group Sham | | |
| --- | --- | --- | --- | --- | --- | --- | --- | --- | --- | --- | --- | --- | --- | --- | --- |
|  | Pre  (SD) | Post  (SD) | P-  value | Pre  (SD) | Post  (SD) | P-  value | Pre  (SD) | Post  (SD) | P-  value | Pre  (SD) | Post  (SD) | P-  value | Pre  (SD) | Post  (SD) | P-  value |
| LF | 0.695  0.0685 | 0.683 0.0726 | 0.524 | 0.671 0.0834 | 0.685 0.0853 | 0.393 | 0.681 0.0880 | 0.682 0.0708 | 0.931 | 0.659 0.0657 | 0.666 0.0768 | 0.672 | 0.667 0.0942 | 0.675 0.0447 | 0.7 |
| RF | 0.713 0.0749 | 0.679 0.0683 | 0.04 | 0.659 0.0861 | 0.682 0.0746 | 0.192 | 0.673 0.0904 | 0.677 0.0740 | 0.367 | 0.655 0.0723 | 0.682 0.0709 | 0.071 | 0.661 0.0967 | 0.669 0.0579 | 0.693 |
| LC | 0.670 0.0790 | 0.639 0.0716 | 0.162 | 0.644 0.0994 | 0.652 0.101 | 0.813 | 0.650 0.108 | 0.654 0.0852 | 0.839 | 0.636 0.0715 | 0.644 0.0982 | 0.75 | 0.644 0.106 | 0.625 0.0969 | 0.737 |
| RC | 0.674 0.0834 | 0.652 0.0918 | 0.281 | 0.641 0.0941 | 0.654 0.111 | 0.614 | 0.662 0.0982 | 0.644 0.101 | 0.365 | 0.644 0.0870 | 0.638 0.0886 | 0.777 | 0.644 0.0783 | 0.642 0.0689 | 0.942 |
| LPO | 0.689 0.0749 | 0.675 0.0526 | 0.429 | 0.653 0.0916 | 0.671 0.0761 | 0.205 | 0.664 0.0772 | 0.661 0.0721 | 0.865 | 0.657 0.0737 | 0.675 0.613 | 0.33 | 0.66 0.102 | 0.667 0.0625 | 0.76 |
| RPO | 0.659 0.0737 | 0.674 0.0847 | 0.451 | 0.651 0.0595 | 0.667 0.0805 | 0.319 | 0.682 0.0928 | 0.683 0.0662 | 0.732 | 0.646 0.729 | 0.666 0.0562 | 0.223 | 0.644 0.105 | 0.675 0.0648 | 0.178 |
| LCE | 0.713 0.0707 | 0.706 0.0628 | 0.655 | 0.687 0.0776 | 0.689 0.0843 | 0.919 | 0.686 0.101 | 0.696 0.0698 | 0.604 | 0.662 0.0659 | 0.685 0.0672 | 0.218 | 0.69 0.0902 | 0.687 0.072 | 0.886 |
| RCE | 0.684 0.0976 | 0.695 0.0750 | 0.501 | 0.678 0.0697 | 0.684 0.0772 | 0.711 | 0.693 0.0835 | 0.696 0.0712 | 0.849 | 0.660 0.0609 | 0.690 0.0658 | 0.05 | 0.675 0.0984 | 0.695 0.055 | 0.358 |

LF: Left frontal lobe, RF: Right frontal lobe, LC: Left central area, RC: Right central area, LPO: Left parieto-occipital lobe, RPO: Right parieto-occipital lobe, LCE: Left cerebellum, RCE: Right cerebellum.

| Theta band | Group 1Hz | | | Group 5Hz | | | Group 10Hz | | | Group 20Hz | | | Group Sham | | |
| --- | --- | --- | --- | --- | --- | --- | --- | --- | --- | --- | --- | --- | --- | --- | --- |
|  | Pre  (SD) | Post  (SD) | P-  value | Pre  (SD) | Post  (SD) | P-  value | Pre  (SD) | Post  (SD) | P-  value | Pre  (SD) | Post  (SD) | P-  value | Pre  (SD) | Post  (SD) | P-  value |
| LF | 0.636±0.071 | 0.662  ±0.060 | 0.353 | 0.635±0.060 | 0.665±0.074 | **0.012** | 0.630±0.091 | 0.646±0.064 | 0.471 | 0.631±0.061 | 0.647±0.066 | 0.464 | 0.627±0.082 | 0.645±0.054 | 0.400 |
| RF | 0.644±0.089 | 0.666  ±0.067 | 0.353 | 0.626±0.053 | 0.668±0.064 | **0.003** | 0.625±0.087 | 0.637±0.063 | 0.471 | 0.635±0.061 | 0.644±0.073 | 0.705 | 0.621±0.059 | 0.639±0.047 | 0.400 |
| LC | 0.579±0.101 | 0.603  ±0.088 | 0.399 | 0.576±0.058 | 0.622±0.089 | **0.027** | 0.590±0.091 | 0.595±0.069 | 0.777 | 0.591±0.060 | 0.595±0.073 | 0.888 | 0.574±0.073 | 0.601±0.058 | 0.400 |
| RC | 0.593±0.098 | 0.574  ±0.098 | 0.399 | 0.554±0.079 | 0.589±0.078 | 0.071 | 0.571±0.096 | 0.598±0.080 | 0.471 | 0.593±0.093 | 0.583±0.084 | 0.888 | 0.568±0.085 | 0.579±0.070 | 0.533 |
| LPO | 0.641±0.080 | 0.661  ±0.081 | 0.353 | 0.622±0.064 | 0.656±0.084 | **0.024** | 0.618±0.092 | 0.636±0.072 | 0.471 | 0.626±0.62 | 0.651±0.066 | 0.388 | 0.625±0.057 | 0.634±0.058 | 0.504 |
| RPO | 0.632±0.786 | 0.660  ±0.068 | 0.265 | 0.619±0.054 | 0.666±0.071 | **0.004** | 0.632±0.096 | 0.648±0.061 | 0.471 | 0.627±0.064 | 0.654±0.061 | 0.388 | 0.619±0.082 | 0.639±0.057 | 0.400 |
| LCE | 0.674±0.080 | 0.689  ±0.061 | 0.353 | 0.657±0.060 | 0.688±0.082 | **0.035** | 0.661±0.089 | 0.683±0.066 | 0.471 | 0.659±0.067 | 0.682±0.072 | 0.388 | 0.658±0.071 | 0.674±0.053 | 0.400 |
| RCE | 0.670±0.081 | 0.693  ±0.062 | 0.353 | 0.663±0.057 | 0.689±0.080 | **0.050** | 0.658±0.092 | 0.684±0.071 | 0.471 | 0.660±0.068 | 0.682±0.072 | 0.388 | 0.659±0.069 | 0.673±0.051 | 0.400 |

Table S4. Local efficiency of each brain region across groups in the theta frequency band before and after rTMS

The p-values in the table are FDR-adjusted p-values. *Significant changes from baseline highlighted in bold. LF: Left frontal lobe, RF: Right frontal lobe, LC: Left central area, RC: Right central area, LPO: Left parieto-occipital lobe, RPO: Right parieto-occipital lobe, LCE: Left cerebellum, RCE: Right cerebellum.

| Alpha band | Group 1Hz | | | Group 5Hz | | | Group 10Hz | | | Group 20Hz | | | Group Sham | | |
| --- | --- | --- | --- | --- | --- | --- | --- | --- | --- | --- | --- | --- | --- | --- | --- |
|  | Pre  (SD) | Post  (SD) | P-  value | Pre  (SD) | Post  (SD) | P-  value | Pre  (SD) | Post  (SD) | P-  value | Pre  (SD) | Post  (SD) | P-  value | Pre  (SD) | Post  (SD) | P-  value |
| LF | 0.739±0.117 | 0.757±0.0975 | 0.348 | 0.768 0.0842 | 0.758 0.0919 | 0.531 | 0.745±0.105 | 0.746±0.0823 | 0.972 | 0.728±0.105 | 0.737±0.0770 | 0.556 | 0.739±0.0888 | 0.760±0.0856 | 0.159 |
| RF | 0.730±0.108 | 0.746±0.0986 | 0.369 | 0.771 0.0897 | 0.744 0.0832 | 0.1 | 0.742±0.104 | 0.741±0.0740 | 0.972 | 0.722±0.103 | 0.723±0.0816 | 0.98 | 0.737±0.0893 | 0.742±0.0853 | 0.723 |
| LC | 0.693±0.125 | 0.682±0.135 | 0.682 | 0.711 0.118 | 0.677 0.0991 | 0.171 | 0.650±0.131 | 0.696±0.969 | 0.023 | 0.681±0.116 | 0.654±0.110 | 0.268 | 0.660±0.121 | 0.677±0.104 | 0.549 |
| RC | 0.661±0.149 | 0.673±0.118 | 0.682 | 0.695 0.145 | 0.673 0.106 | 0.192 | 0.669±0.139 | 0.660±0.0981 | 0.713 | 0.666±0.113 | 0.649±0.118 | 0.426 | 0.651±0.114 | 0.690±0.114 | 0.165 |
| LPO | 0.701±0.125 | 0.715±0.109 | 0.468 | 0.744 0.0891 | 0.719 0.0985 | 0.141 | 0.709±0.104 | 0.717±0.0677 | 0.631 | 0.703±0.0997 | 0.712±0.0847 | 0.587 | 0.714±0.0871 | 0.727±0.0956 | 0.399 |
| RO | 0.724±0.107 | 0.744±0.0984 | 0.289 | 0.755 0.0839 | 0.744 0.0934 | 0.555 | 0.731±0.107 | 0.729±0.0762 | 0.899 | 0.720±0.102 | 0.735±0.0785 | 0.422 | 0.729±0.0902 | 0.745±0.0912 | 0.337 |
| LCE | 0.747±0.100 | 0.766±0.0932 | 0.255 | 0.767 0.0920 | 0.761 0.0922 | 0.728 | 0.756±0.0986 | 0.755±0.0771 | 0.937 | 0.721±0.107 | 0.745±0.0803 | 0.107 | 0.739±0.0668 | 0.767±0.0896 | 0.062 |
| RCE | 0.743±0.0994 | 0.762±0.0898 | 0.256 | 0.757 0.115 | 0.754 0.0834 | 0.658 | 0.751±0.106 | 0.751±0.0745 | 0.984 | 0.733±0.109 | 0.736±0.0816 | 0.754 | 0.740±0.0904 | 0.764±0.0797 | 0.212 |

Table S5. Local efficiency of each brain region across groups in the alpha frequency band before and after rTMS

LF: Left frontal lobe, RF: Right frontal lobe, LC: Left central area, RC: Right central area, LPO: Left parieto-occipital lobe, RPO: Right parieto-occipital lobe, LCE: Left cerebellum, RCE: Right cerebellum.

Table S6. Local efficiency of each brain region across groups in the beta1 frequency band before and after rTMS

| Beta1 band | Group 1Hz | | | Group 5Hz | | | Group 10Hz | | | Group 20Hz | | | Group Sham | | |
| --- | --- | --- | --- | --- | --- | --- | --- | --- | --- | --- | --- | --- | --- | --- | --- |
|  | Pre  (SD) | Post  (SD) | P-  value | Pre  (SD) | Post  (SD) | P-  value | Pre  (SD) | Post  (SD) | P-  value | Pre  (SD) | Post  (SD) | P-  value | Pre  (SD) | Post  (SD) | P-  value |
| LF | 0.629 0.0745 | 0.637 0.0677 | 0.357 | 0.630 0.0845 | 0.630 0.0721 | 0.977 | 0.623 0.0643 | 0.626 0.0659 | 0.84 | 0.595 0.0675 | 0.618 0.0695 | 0.125 | 0.622 0.0649 | 0.631 0.0588 | 0.577 |
| RF | 0.626 0.0735 | 0.632 0.0635 | 0.518 | 0.628 0.093 | 0.626 0.0713 | 0.936 | 0.623 0.0618 | 0.622 0.0663 | 0.918 | 0.602 0.0676 | 0.610 0.0609 | 0.549 | 0.619 0.0649 | 0.633 0.0632 | 0.384 |
| LC | 0.565 0.0587 | 0.571 0.0930 | 0.735 | 0.565 0.107 | 0.571 0.071 | 0.72 | 0.574 0.0645 | 0.55 0.0565 | 0.098 | 0.552 0.0576 | 0.559 0.0622 | 0.905 | 0.581 0.0678 | 0.558 0.0432 | 0.121 |
| RC | 0.575 0.0675 | 0.573 0.0855 | 0.903 | 0.565 0.0945 | 0.564 0.079 | 0.92 | 0.559 0.0532 | 0.573 0.0491 | 0.281 | 0.541 0.0570 | 0.569 0.0477 | 0.044 | 0.566 0.0483 | 0.556 0.0813 | 0.531 |
| LPO | 0.620 0.0715 | 0.595 0.0563 | 0.066 | 0.616 0.0886 | 0.614 0.0724 | 0.838 | 0.613 0.0665 | 0.614 0.0608 | 0.937 | 0.590 0.600 | 0.598 0.0541 | 0.567 | 0.617 0.0599 | 0.623 0.0689 | 0.668 |
| RPO | 0.623 0.0689 | 0.622 0.0744 | 0.907 | 0.620 0.0738 | 0.623 0.0703 | 0.873 | 0.615 0.0675 | 0.621 0.0609 | 0.689 | 0.590 0.0685 | 0.614 0.0649 | 0.088 | 0.615 0.0636 | 0.613 0.0543 | 0.861 |
| LCE | 0.623 0.0751 | 0.629 0.0998 | 0.703 | 0.643 0.0895 | 0.635 0.0732 | 0.645 | 0.637 0.0613 | 0.649 0.063 | 0.499 | 0.614 0.0757 | 0.630 0.0728 | 0.456 | 0.633 0.06 | 0.637 0.0697 | 0.828 |
| RCE | 0.620 0.0638 | 0.642 0.0768 | 0.135 | 0.630 0.0739 | 0.643 0.076 | 0.46 | 0.630 0.0661 | 0.653 0.067 | 0.239 | 0.609 0.0662 | 0.627 0.0632 | 0.253 | 0.622 0.0536 | 0.637 0.0811 | 0.369 |

LF: Left frontal lobe, RF: Right frontal lobe, LC: Left central area, RC: Right central area, LPO: Left parieto-occipital lobe, RPO: Right parieto-occipital lobe, LCE: Left cerebellum, RCE: Right cerebellum.

1. **Correlation analysis**

To further explore the relationship between ERP and oscillatory activity, we performed a correlation analysis using the difference amplitude and difference power (Δ). The results presented in Figure S7 demonstrate that in the 5 Hz rTMS group, P150 showed significant positive correlations with theta (r=0.557, P=0.001), alpha (r=0.679, P=0.000), and beta1 (r=0.544, P=0.002). This indicates that after stimulation, higher amplitudes of P150 are associated with increased power in the theta, alpha, and beta1 frequency bands (Figure S7).


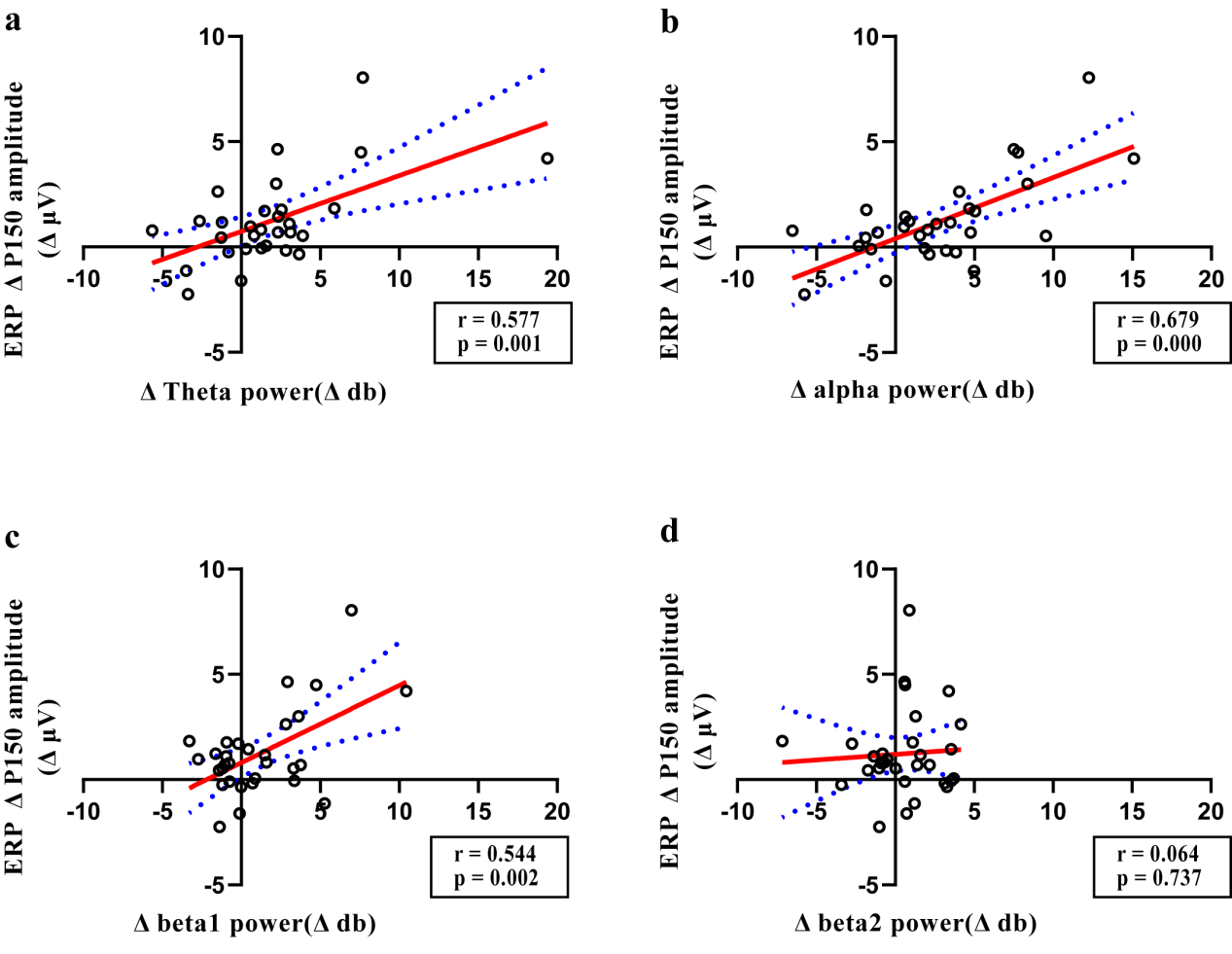


Figure S7. Correlation between the average amplitude of each ERP (N100, P150, N200, P300) and oscillatory activity (theta, alpha, beta1, and beta2) in the rTMS5 group during the 2-back task

**References**

[1] Delorme A, Makeig S. EEGLAB: an open source toolbox for analysis of single-trial EEG dynamics including independent component analysis. J Neurosci Methods 2004;134(1):9-21.

[2] Yao Q, Tang F, Wang Y, Yan Y, Dong L, Wang T, et al. Effect of cerebellum stimulation on cognitive recovery in patients with Alzheimer disease: A randomized clinical trial. Brain Stimulation 2022;15(4):910-20.

[3] Cracco RQ, Amassian VE, Maccabee PJ, Cracco JB. Comparison of human transcallosal responses evoked by magnetic coil and electrical stimulation. Electroencephalogr Clin Neurophysiol 1989;74(6):417-24.

[4] Aydore S, Pantazis D, Leahy RM. A note on the phase locking value and its properties. Neuroimage 2013;74:231-44.

[5] Li Y, Yang B, Wang Z, Huang R, Lu X, Bi X, et al. EEG assessment of brain dysfunction for patients with chronic primary pain and depression under auditory oddball task. Front Neurosci 2023;17:1133834.
